# Pre-pregnancy and early pregnancy dietary patterns and gestational diabetes risk among Miao women in China

**DOI:** 10.3389/fnut.2025.1663054

**Published:** 2026-01-06

**Authors:** Song Zhang, Xiaorong Ni, Tian Qiao, Danqing Zhao, Liming Shen, Yi Liang

**Affiliations:** 1 Department of Endocrinology, Affiliated Hospital of Guizhou Medical University, Guiyang, China; 2 Department of Clinical Nutrition, Shenzhen Hengsheng Hospital, Shenzhen, China; 3 Department of Clinical Nutrition, Affiliated Hospital of Guizhou Medical University, Guiyang, China; 4 Obstetrics, Affiliated Hospital of Guizhou Medical University, Guiyang, China; 5 College of Life Science and Oceanography, Shenzhen University, Shenzhen, China

**Keywords:** dietary patterns, gestational diabetes mellitus (GDM), prospective cohort, pregnant women, Chinese Miao ethnicity

## Abstract

**Background:**

Gestational diabetes mellitus (GDM) affects 5–17% of pregnancies globally. However, research on the relationship between dietary patterns and GDM risk is scarce in Asia, especially among ethnic minority groups. This study explored the links between pre-pregnancy and early pregnancy dietary patterns and the risk of GDM in Miao pregnant women in China.

**Methods:**

In this prospective cohort study, we recruited 683 Miao pregnant women and assessed dietary intake using validated food frequency questionnaires covering the year before conception and early pregnancy. Principal component analysis identified major dietary patterns, and multivariable logistic regression models evaluated associations with GDM risk. Restricted cubic spline analysis examined dose-response relationships between traditional Miao foods and GDM.

**Results:**

Among participants, 130 women (19.03%) developed GDM. Two distinct dietary patterns emerged: a “prudent” pattern (whole grains, vegetables, fruits, beans, nonprocessed meat, eggs) and a “processed” pattern (processed meat, snacks, convenience foods, dessert, beverages). Higher adherence to the pre-pregnancy prudent pattern was associated with significantly reduced GDM odds (highest vs. lowest quartile: OR: 0.49; 95% CI: 0.24, 0.97; *P*-trend=0.049). Similarly, early pregnancy adherence to the prudent pattern demonstrated a significant inverse association with GDM risk in fully adjusted models, with the highest quartile showing a 56% reduction in risk (OR: 0.44; 95% CI: 0.22, 0.89; *P*-trend = 0.031). No significant association was observed between the processed pattern and GDM risk after adjustment for potential confounders. Sour soup consumption exhibited protective associations during both study periods, with significant overall associations during preconception (*P* = 0.044) and early pregnancy (*P* = 0.011).

**Conclusions:**

Adherence to a prudent dietary pattern during preconception and early pregnancy is associated with a reduction in GDM risk among Miao women. Miao traditional sour soup was found to protect against GDM. These findings suggest that promoting healthy dietary habits, particularly focusing on traditional dietary practices, may be an effective strategy for reducing GDM risk in this population.

## 1 Introduction

Gestational diabetes mellitus (GDM), characterized by glucose intolerance first detected during pregnancy, has emerged as a significant global health concern affecting 5–17% of pregnancies worldwide over the past two decades (1, 2). This upward trend is projected to continue due to increasing rates of overweight and obesity among women of reproductive age (3–5). GDM poses significant risks, including preeclampsia (6) and subsequent type 2 diabetes in mothers (7), while offspring face increased risks of macrosomia (8), obesity (9), and future metabolic disorders (10). Among the constellation of risk factors associated with GDM development, dietary factors represent a critical modifiable component for prevention strategies. Evidence from nutritional epidemiology demonstrates that specific nutrient profiles including—low intake of polyunsaturated fatty acids (11), fiber (12), and low glycemic load foods (13), alongside high consumption of total fat (14), heme iron (15), cholesterol (16), and red/processed meats (17)—significantly increase GDM risk. However, examining isolated nutrients fails to capture the complexity of dietary behaviors, as foods and nutrients are consumed in combination rather than independently (18). Consequently, analysis of comprehensive dietary patterns offers more meaningful insights by accounting for nutrient interactions and cumulative effects.

Recent investigations have established inverse associations between GDM risk and adherence to Mediterranean, DASH, and prudent dietary patterns (19–21), whereas Western dietary patterns correlate positively with GDM incidence (22). In China, limited studies from Guangdong, Hebei, and Shaanxi have identified protective effects from vegetable-rich and deep-sea fish dietary patterns, while patterns characterized by sweets and refined carbohydrates appear to increase GDM risk (23–25). However, there has been no exploration of dietary patterns among ethnic minorities in China who maintain unique culinary traditions. The Miao ethnic group is the fifth-largest minority group in China, with a population of 11.07 million (26), and exhibits distinctive dietary preferences. Their diet centers on polished rice as the staple, featuring common dishes like sour soup, pickled foods, and a range of stews (27). Given the established ethnic disparities in GDM prevalence and the fact that dietary patterns are population-specific, influenced by sociocultural factors and food availability (28), a significant research gap remains regarding the relationship between dietary patterns and GDM risk, particularly among Asian ethnic groups. This prospective cohort study examines associations between dietary patterns (one year pre-pregnancy and early pregnancy) and subsequent GDM risk. We focused on an understudied cohort of Miao women in Guizhou, China, to provide an evidence base for culturally appropriate preventive strategies.

## 2 Materials and methods

### 2.1 . Study population

This prospective investigation utilized data from the Chinese Miao Mother and Child Cohort(MCCMC), established in September 2022, to examine associations between dietary factors and adverse pregnancy outcomes. Participants were recruited from three hospital sites within the cohort center located in Miao settlement areas of Qiandongnan Miao and Dong Autonomous Prefecture, Guizhou Province, China. Pregnant women were eligible for inclusion if they met the following criteria: (a) age ≥18 years, (b) singleton pregnancy, (c) Gestational age ≤ 12 weeks, (d) Miao ethnicity, and permanent residence in the cohort center region for ≥1 year. Women were excluded if they had a history of pre-pregnancy diabetes or fasting blood glucose ≥7.0 mmol/L, prior diagnosis of GDM during previous pregnancy, severe liver or kidney disease, autoimmune diseases, or long-term use of glucocorticoids or other medications that affect glucose metabolism.

Sample size calculation was based on an observed GDM prevalence of 18.0% in southwest China(29), with a significance level (α) of 0.05, a statistical power of 90%, and an allowable margin of error (d) of 3.0%. The required sample size was therefore estimated to be 630 participants. Initially, 730 participants were recruited; 32 were excluded due to loss to follow-up, and 7 were excluded due to incomplete dietary data. During the analysis phase, we further excluded participants whose reported dietary intake reflected implausible total energy intake (< 2.09 MJ [500 kcal]/day or >20.92 MJ [5,000 kcal]/day) to minimize dietary measurement error.

This study was approved by the Ethics Committee of the Affiliated Hospital of Guizhou Medical University (approval number: 2021 [065-01]), and written informed consent was obtained from all participants prior to enrollment.

### 2.2 Dietary assessment

This study employed a prospective cohort design to conduct longitudinal follow-up surveys among pregnant women. The baseline assessment, conducted during early pregnancy (< 12 weeks gestation), gathered participant information and recalled dietary and physical activity status from the preceding year. A follow-up assessment was then conducted during mid-pregnancy (13 weeks until GDM outcome) to capture dietary intake and physical activity during early pregnancy.

Dietary intake was evaluated using a validated semi-quantitative FFQ comprising 74 food items. The FFQ was specifically designed to capture both common foods consumed in southwestern China and traditional Miao ethnic cuisine, including sour soup (red and white varieties), cured pork, pickled vegetables, and others. This instrument has demonstrated reliable validity and reproducibility in previous validation studies among Miao pregnant women (30).

Data were gathered via face-to-face interviews conducted by trained investigators. Participants reported their consumption frequency (daily, weekly, monthly, or never) for each food item. When participants provided frequency ranges (e.g., “4–5 times/week”), the median value (4.5 times/week) was recorded.

More accurate portion size estimation was facilitated using standardized food models and food maps as visual references. The models represented a standard 90 kcal portion, and participant intake was recorded as multiples thereof (e.g., 0.5 × , 2 × ). For items lacking physical models, food maps were employed in conjunction with a validated conversion table. This table established weight equivalents for all depicted portions (e.g., 1 plate of shredded potatoes = 100 g) and was developed based on pre-study consultations with local nutritionists and market surveys. Daily food intake (g/day) was calculated by multiplying the daily consumption frequency by the portion weight (g). Nutrient intakes were determined by multiplying daily food intake by the corresponding nutrient density values from the Chinese Food Composition Table (31). Nutrient calculations excluded sour soup, a traditional Miao food, due to limited available data on its detailed nutritional composition, despite studies examining its microbial community diversity (32, 33).

### 2.3 Non-dietary covariates

Multiple non-dietary covariates were evaluated as potential confounding factors, including maternal demographics, physical activity, obstetric and gynecological history, and familial diabetes. Pre-pregnancy body mass index (BMI) [kg/m^2^] was calculated based on weight and height measurements taken on-site. Parity was classified as either nulliparous (0 previous births) or multiparous (≥1 previous births). Family history of diabetes was recorded as positive when participants reported diabetes diagnoses in immediate family members (parents or siblings). The history of polycystic ovary syndrome (PCOS) was documented as yes or no. The International Physical Activity Questionnaire (IPAQ) was used to measure participants' intensity and duration of weekly physical activity during preconception and early pregnancy.

### 2.4 Ascertainment of GDM

A standardized 2-h 75g oral glucose tolerance test (OGTT) was administered between the 24th and 28th weeks of gestation to screen for GDM. All OGTT results were documented in the electronic medical record systems of the hospital sites within the cohort center. GDM was ascertained if they met at least one of the following criteria: fasting plasma glucose >5.1 mmol/L, 1-h post-load glucose >10.0 mmol/L, or 2-h post-load glucose ≥8.5 mmol/L based on the 2011 criteria established by the Ministry of Health of China (34). For participants who underwent OGTT screening at facilities outside the cohort center, GDM status was ascertained through telephone follow-up interviews. In cases where participants self-reported a GDM diagnosis but complete OGTT values (all three time points) could not be obtained, we recorded the binary outcome of physician-diagnosed GDM without the specific glucose measurements.

### 2.5 Statistical analysis

We classified 74 foods into 23 food groups based on nutritional similarities. Sour soup was categorized separately due to its distinctive nutrient profiles. Principal component analysis with varimax rotation was used to identify dietary patterns. The Kaiser–Meyer–Olkin test (KMO=0.749 for pre-pregnancy; KMO = 0.722 for early pregnancy) and the Bartlett test of sphericity (all P < 0.001) suggested that the data structure was reasonable. Eventually, we determined the number of patterns using scree plot analysis, eigenvalues (>1), factor interpretability, and explained variance. Food categories contributing to each dietary pattern were determined based on factor loadings ≥0.3. Subsequently, Principal component scores were calculated for each pregnant woman, with the highest value representing the pregnant woman's dietary pattern. For each dietary pattern, the higher the principal component score, the higher the degree of adherence to the pattern. Participants were divided into quartiles (Q1-Q4) according to their dietary pattern scores. Continuous variables are presented as median and interquartile range (Median ± IQR), and comparisons between groups were made using the Wilcoxon rank-sum test. Categorical variables are presented as frequency and percentage [*n*(%)].

Logistic regression models were used to assess the association between dietary patterns and the risk of GDM, with odds ratios (OR) and 95% confidence intervals (95% CI) calculated. In the multivariate analysis, model 1 was the unadjusted model; model 2 adjusted for other dietary patterns, maternal age, household monthly income per capita, occupation, and family history of PCOS; model 3 further adjusted for pre-pregnancy BMI, physical activity level, and energy intake (quartiles) based on model 2. To assess linear trends, quartile groups for each dietary pattern were treated as continuous variables and included in logistic regression models to calculate P trend values. Additionally, we employed restricted cubic spline analyses to examine potential dose-response relationships between consumption of traditional Miao foods (including sour soup and pickled vegetables) and GDM.

## 3 Results

### 3.1 General characteristics of participants

In this prospective cohort study of 683 Miao women, 130 participants (19.03%) developed GDM (Table 1). The mean age of the cohort at enrollment was 30.44 ± 4.76 years. The largest age group was 30–35 years, accounting for 275 (40.26%) of the participants. Most participants (652 [95.46%]) were married. Educational attainment varied considerably: 405 (59.30%) had completed 13-15 years of formal education, while 193 (28.26%) had less than 9 years of schooling. The mean pre-pregnancy BMI was 22.36 ± 3.49. 423 (61.93%) participants were classified as normal weight, 184 (26.94%) as overweight or obese, and 76 (11.13%) as underweight. Regarding occupation, 101 (14.79%) participants were manual workers. More than half of the women (354 [51.83%]) had multiple pregnancies. Medical history included PCOS in 29 (4.25%) participants and a family history of diabetes in 45 (6.59%). Physical activity was predominantly of light intensity during.

**Table 1:** Participants' characteristics in the present study (*n* = 683).

**Baseline**	**Group**	***n***	**%**
Age at enrollment (y)	18–24	56	8.20
	25–29	252	36.90
	30–35	275	40.26
	≥35	100	14.64
Marital status (married)		652	95.46
Schooling years	≤ 9	193	28.26
	9–12	80	11.71
	13–15	405	59.3
	≥16	5	0.73
Pre-pregnancy BMI	< 18.5	76	11.13
	18.5–23.9	423	61.93
	≥24	184	26.94
Occupation (manual worker)		101	14.79
Household monthly income per capita	< 1,000 CNY	20	2.93
	1,000–3,000 CNY	220	32.21
	≥3,000 CNY	443	64.86
Parity (≥1, %)		354	51.83
PCOS history (yes, %)		29	4.25
GDM (yes, %)		130	19.03
Family history of diabetes (yes, %)		45	6.59
Weekly physical activity intensity^a^ [pre/early pregnancy]	Might	513/601	75.11/87.99
	Moderate	62/44	9.10/6.44
	High	108/38	15.81/5.56

^a^Assessment of pregnant women's weekly physical activity levels utilizing the International Physical Activity Questionnaire (IPAQ).

### 3.2 Dietary patterns among Miao pregnant women during preconception and early pregnancy periods

**Figure 1:**
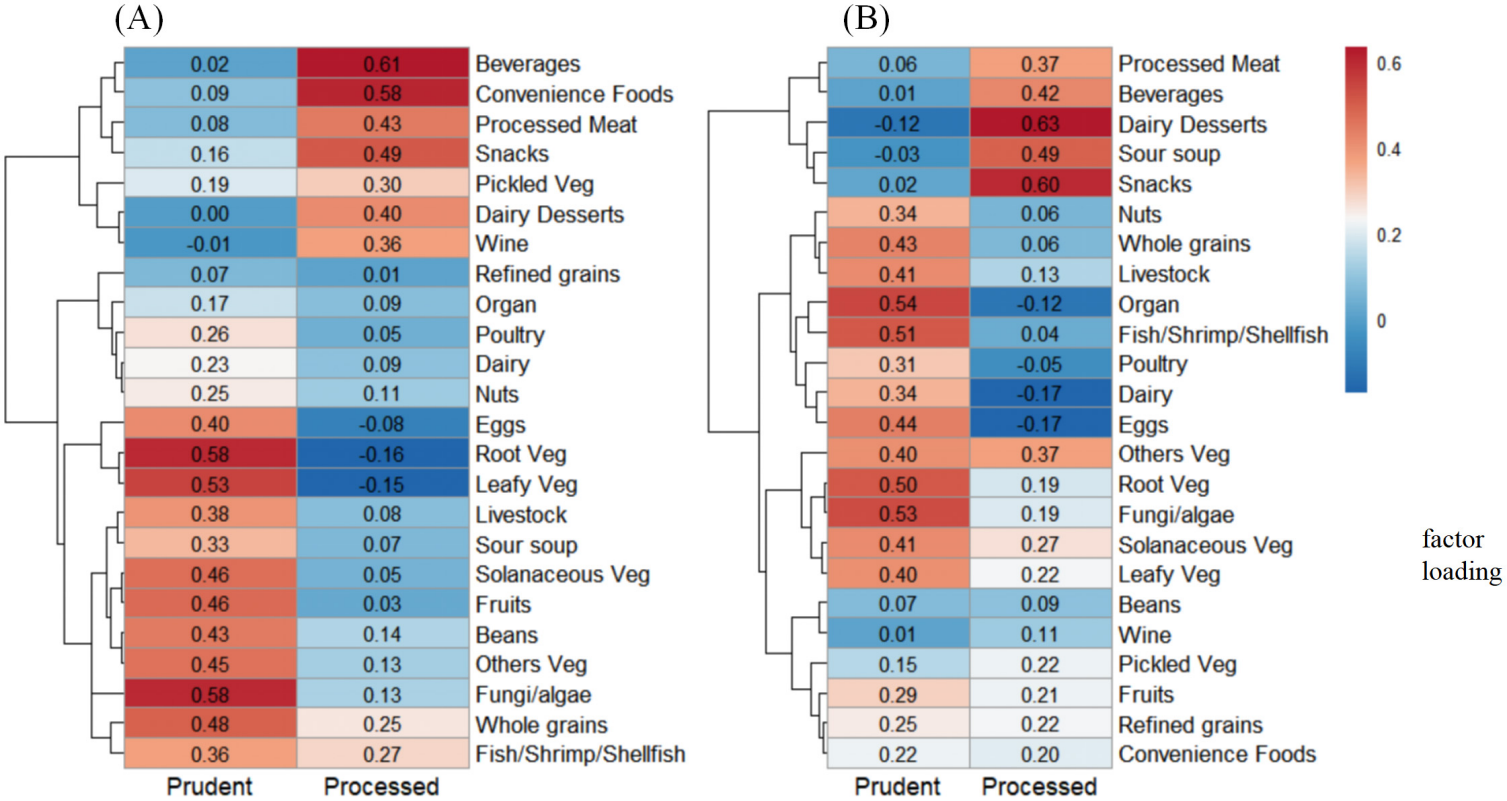
Cluster diagram of factor loadings of food group for two dietary patterns identified among Miao pregnant women. Panels **(A)** and **(B)** represent the preconception year and early pregnancy periods, respectively. The numerical values in the heatmaps represent factor loadings, which indicate the strength and direction of association between each food group and the dietary pattern. The closer the absolute value of the factor loading is to 1, the greater its contribution to the principal component. The hierarchical clustering dendrogram on the left side of each heatmap groups food items with similar loading patterns.

Principal component analysis identified two major dietary patterns among Miao women during both preconception and early pregnancy periods (Supplementary Table S1). The first pattern, characterized by high consumption of whole grains, vegetables, fungi/algae, fruits, beans, meat, and eggs, was designated the “prudent” pattern. The second pattern, featuring processed meats, snacks, convenience foods, desserts, and beverages, was termed the “processed” pattern. These patterns explained 11.78% and 7.58% of dietary variance during preconception, and 11.25% and 8.13% during early pregnancy, respectively. Similar clustering was observed in hierarchical analysis (Figure 1).

Women with higher adherence to the prudent pattern consumed significantly greater quantities of vegetables, fungi/algae, beans, meat, and eggs during both periods compared with those following the processed pattern (all *P* < 0.05; Supplementary Table S2). Conversely, processed pattern adherents had significantly higher intakes of processed meats, pickled vegetables, snacks, convenience food, dairy desserts, and beverages (all *P* < 0.05). Nutrient analysis revealed that prudent pattern followers had significantly higher intakes of protein, fatty acids, dietary fiber, vitamins, and minerals during both preconception and early pregnancy periods compared with the processed pattern group (all *P* < 0.05; Table 2).

**Table 2:** Comparison of nutrient intake across different dietary patterns among Miao pregnant women during the year before conception and early pregnancy^b^.

**Nutrients**	**One year preconception**		***P* value**	**Early pregnancy**		***P* value**
	**Prudent**	**Processed**		**Prudent**	**Processed**	
*n*	329	354		345	338	
Energy, kcal d ^−1^	2,011.26 ± 973.71	2,017.46 ± 958.62	0.916	1,962.05 ± 870.04^a^	1,699.62 ± 864.57	< 0.001
Protein, g d^−1^	69.76 ± 36.59^a^	59.29 ± 31.29	< 0.001	72.24 ± 36.16^a^	49.57 ± 31.17	< 0.001
Fat, g d^−1^	82.32 ± 48.20	84.24 ± 46.32	0.966	82.26 ± 47.12^a^	66.67 ± 39.53	< 0.001
SFA, g d^−1^	25.09 ± 17.52^a^	23.48 ± 14.07	0.002	25.82 ± 14.77^a^	20.19 ± 11.69	< 0.001
MUFA, g d^−1^	28.72 ± 18.86^a^	26.74 ± 16.24	0.002	28.89 ± 18.26^a^	22.46 ± 13.46	< 0.001
PUFA, g d^−1^	12.06 ± 5.14^a^	11.57 ± 5.11	0.035	14.00 ± 7.55^a^	11.13 ± 5.69	< 0.001
Carbohydrate, g d^−1^	246.09 ± 114.32	249.97 ± 135.66	0.187	237.37 ± 100.97	225.91 ± 118.65	0.123
Cholesterol,g d^−1^	428.20 ± 332.63^a^	280.65 ± 230.54	< 0.001	515.96 ± 295.80^a^	252.98 ± 247.55	< 0.001
Fiber,g d^−1^	15.07 ± 9.20^a^	10.42 ± 6.20	< 0.001	15.20 ± 9.58^a^	12.66 ± 8.29	< 0.001
Vitamin A, ug d^−1^	1620.87 ± 1595.29^a^	960.76 ± 835.16	< 0.001	1405.05 ± 1203.78^a^	960.59 ± 1028.81	< 0.001
Thiamine, mg d^−1^	0.89 ± 0.60^a^	0.74 ± 0.51	< 0.001	0.88 ± 0.51^a^	0.63 ± 0.43	< 0.001
Riboflavin, mg d^−1^	1.12 ± 0.56^a^	0.87 ± 0.54	< 0.001	1.20 ± 0.50^a^	0.77 ± 0.52	< 0.001
Niacin, mg d^−1^	18.83 ± 11.02^a^	16.68 ± 11.11	0.013	16.67 ± 9.70^a^	12.48 ± 8.26	< 0.001
Vitamin B6, mg d^−1^	0.17 ± 0.13^a^	0.13 ± 0.13	< 0.001	0.16 ± 0.14^a^	0.13 ± 0.12	< 0.001
Vitamin D, mg d^−1^	1.90 ± 2.58^a^	1.31 ± 1.83	< 0.001	3.88 ± 1.82^a^	1.77 ± 1.96	< 0.001
Vitamin C, mg d^−1^	689.18 ± 1097.00^a^	477.40 ± 856.78	< 0.001	526.23 ± 844.94	557.49 ± 927.43	0.813
Vitamin E, mg d^−1^	23.16 ± 11.11^a^	21.38 ± 10.21	< 0.001	25.91 ± 12.68^a^	21.74 ± 12.28	< 0.001
Folic acid, ug d^−1^	55.01 ± 53.56^a^	33.33 ± 33.85	< 0.001	46.33 ± 51.78^a^	35.05 ± 43.17	< 0.001
Carotene, mg d^−1^	7822.23 ± 6834.84^a^	4295.35 ± 3395.95	< 0.001	5766.22 ± 5566.39^a^	4756.66 ± 4799.04	< 0.001
Calcium, mg d^−1^	714.80 ± 444.85^a^	537.42 ± 377.00	< 0.001	790.24 ± 396.22^a^	550.51 ± 429.80	< 0.001
Potassium, mg d^−1^	2850.05 ± 1697.54^a^	2447.01 ± 1647.56	< 0.001	2833.80 ± 1385.44^a^	2217.03 ± 1475.39	< 0.001
Magnesium, mg d^−1^	339.63 ± 185.67^a^	264.44 ± 144.61	< 0.001	329.35 ± 191.25^a^	257.66 ± 176.22	< 0.001
Iron, mg d^−1^	20.64 ± 11.00^a^	16.46 ± 8.96	< 0.001	20.25 ± 11.25^a^	16.08 ± 11.75	< 0.001
Sodium, mg d^−1^	10542.82 ± 4431.85	10757.01 ± 5985.34	0.977	10887.29 ± 4626.84	10135.64 ± 5464.29^a^	0.012
Iodine, mg d^−1^	1.41 ± 1.54	1.71 ± 2.03^a^	0.002	2.05 ± 2.04^a^	1.74 ± 2.17	0.002

^b^ Unless otherwise specified, data are presented as median ± interquartile range (IQR). *P*-values were calculated using the Wilcoxon rank sum test. ^a^Indicates significantly higher intake in this dietary pattern group (*P* < 0.05).

### 3.3 Association between dietary patterns and GDM

Table 3 presents the odds ratios of GDM according to quartiles of dietary pattern scores during the preconception year and early pregnancy. For the prudent dietary pattern during preconception, a significant inverse association was observed with GDM risk in the fully adjusted model (Model 3), with the highest quartile showing a 51% reduced risk (OR: 0.49; 95% CI: 0.24, 0.97) compared to the lowest quartile (*P* for trend = 0.049). Similarly, during the early pregnancy, adherence to the prudent dietary pattern was significantly associated with lower GDM risk in the fully adjusted model, with the highest quartile demonstrating a 56% reduction in risk (OR: 0.44; 95% CI: 0.22, 0.89; *P* for trend = 0.031). However, we did not observe a significant association between processed dietary prudent and GDM after adjusting for other confounding variables, either in the year before pregnancy or during early pregnancy.

**Table 3:** Odds ratio of GDM (with 95% CIs) according to quartiles of one year preconception and first trimester dietary pattern scores.^a, b^.

**Models**	**Quartiles of dietary pattern scores**				***P* for trend**
**One year preconception**					
**Prudent**					
Model 1	1 (Reference)	1.00 (0.59, 1.70)	1.04 (0.62, 1.77)	0.76 (0.43, 1.32)	0.388
Model 2	1 (Reference)	0.90 (0.52, 1.56)	0.93 (0.53, 1.62)	0.72 (0.40, 1.28)	0.370
Model 3	1 (Reference)	0.76 (0.42, 1.38)	0.72 (0.38, 1.37)	0.49 (0.24, 0.97)	0.049
**Processed**					
Model 1	1 (Reference)	0.66 (0.38, 1.13)	0.94 (0.56, 1.57)	0.61(0.35, 1.0)	0.195
Model 2	1 (Reference)	0.64 (0.36, 1.12)	1.03 (0.60, 1.77)	0.71 (0.40, 1.26)	0.545
Model 3	1 (Reference)	0.69 (0.38, 1.24)	1.12 (0.64, 1.96)	0.76 (0.41, 1.41)	0.758
**Early pregnancy**					
**Prudent**					
Model 1	1 (Reference)	0.81 (0.48, 1.36)	0.78 (0.46, 1.32)	0.61 (0.35, 1.05)	0.084
Model 2	1 (Reference)	0.76 (0.44, 1.31)	0.84 (0.48, 1.44)	0.66 (0.37, 1.16)	0.228
Model 3	1 (Reference)	0.62 (0.34, 1.11)	0.58 (0.31, 1.08)	0.44 (0.22, 0.89)	0.031
**Processed**					
Model 1	1 (Reference)	0.63 (0.37, 1.07)	0.66 (0.39, 1.11)	0.53 (0.31, 0.91)	0.031
Model 2	1 (Reference)	0.63 (0.36, 1.07)	0.68 (0.39, 1.18)	0.52 (0.29, 0.93)	0.041
Model 3	1 (Reference)	0.66 (0.37, 1.15)	0.77 (0.43, 1.36)	0.60 (0.32, 1.10)	0.151

^a^OR and 95% CI were calculated using logistic regression.^b^Model 1 is a crude model. Model 2 was adjusted for other dietary patterns, maternal age, schooling years, household monthly income per capita, occupation, history of PCOS, and parity. Model 3 was adjusted for Model 2 + family history of diabetes, pre-pregnancy BMI, physical activity level, and total energy (quintile).

### 3.4 Dose-response relationships between traditional Miao food consumption and GDM risk

**Figure 2:**
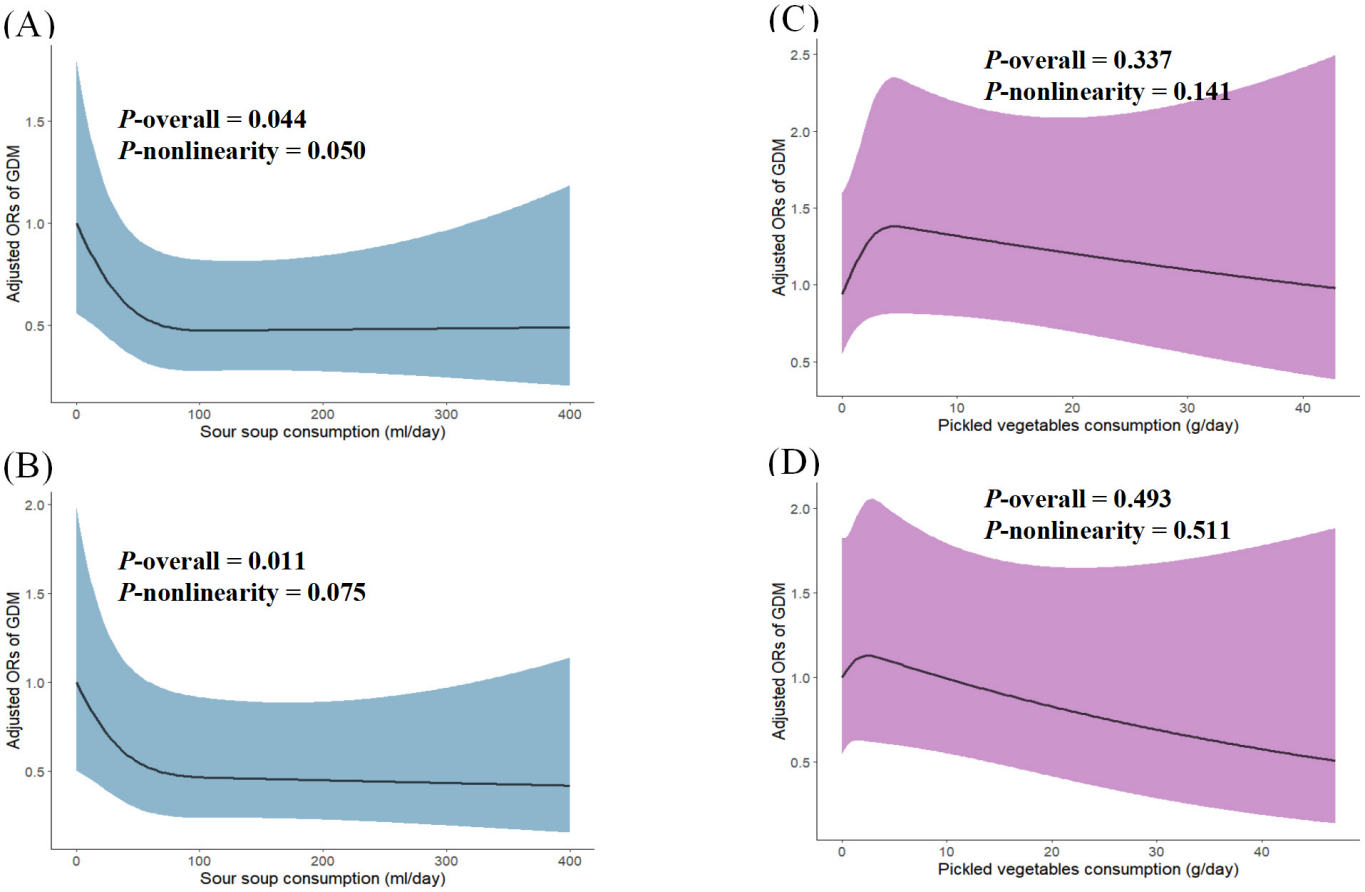
Dose-response relationship between traditional Miao foods and gestational diabetes mellitus (GDM) risk across different time periods using restricted cubic splines. **(A, B)** show the relationship between sour soup consumption and GDM during the year before conception and early pregnancy, respectively. **(C, D)** present the relationship between pickled vegetable consumption and GDM during the same respective time periods. The solid line and blue shading represent the estimated odds ratios (ORs) and their 95% confidence intervals (CIs). Models were adjusted for maternal age, education, occupation, income, family history of diabetes, parity, pre-pregnancy BMI, physical activity, and total energy intake.

Restricted cubic spline analysis revealed distinct dose-response patterns for traditional Miao foods and GDM risk (Figure 2). Sour soup consumption exhibited protective associations during both study periods, with significant overall associations during preconception (*P*-overall = 0.044) and early pregnancy (*P*-overall = 0.011). The association during preconception approached statistical significance for linearity (*P* = 0.050 for non-linearity), while the relationship in early pregnancy exhibited a linear pattern (*P* = 0.073 for non-linearity). By contrast, pickled vegetable consumption was not significantly associated with GDM risk during either the preconception (*P*-overall = 0.337) or early pregnancy (*P*-overall = 0.493) periods. The trends for both periods were linear (*P* for non-linearity = 0.141 and 0.511, respectively).

## 4 Discussion

This study analyzed dietary patterns among Miao pregnant women in Guizhou Province during preconception and early pregnancy. Using the principal component analysis, We identified two main patterns: 'prudent' and 'processed', and investigated their associations with GDM. To our knowledge, this is the first study to systematically characterize dietary patterns in pregnant Miao women using principal component analysis. The prudent pattern—high in vegetables, fruits, legumes, and unprocessed animal foods—that resembles healthy patterns in other Chinese (35) and Western cohorts (36). However, this pattern retained unique Miao ethnic characteristics, notably the inclusion of locally foraged fungi/algae and traditional sour soup. The processed pattern, characterized by processed meats, snacks, convenience foods, and beverages, resembled typical Western dietary patterns (37, 38). Moreover, our findings indicate that adherence to a prudent dietary pattern during the preconception and early pregnancy stages is negatively associated with GDM risk. This protective association aligns with findings from the US Nurses' Health Study cohort, wherein a prudent dietary pattern characterized by high intakes of fruits, green leafy vegetables, poultry, and fish was associated with reduced GDM risk (37). Similarly, Tryggvadottir et al. (21) observed a 54% reduction in GDM risk among Icelandic women following a comparable dietary pattern. Furthermore, adherence to the Mediterranean diet during pregnancy has been shown to reduce GDM risk by up to 80%, while strict adherence to the DASH diet can lower the risk by 71%(19). A systematic review also indicated that healthy dietary patterns can reduce GDM risk by 13–67% (38). Despite methodological variations across studies—including differences in analytical approaches, sample sizes, and cultural contexts—protective dietary patterns consistently share common features: a high intake of fruits and vegetables, limited consumption of red and processed meats, and a focus on the quality rather than the quantity of carbohydrates.

Several biological mechanisms may explain why healthy dietary patterns protect against GDM. Fruits and vegetables are rich in antioxidants (such as vitamins C and E), plant compounds (carotenoids), and dietary fiber. These nutrients help reduce cellular damage and maintain the body's ability to regulate blood sugar effectively during pregnancy's increased metabolic demands (39, 40). Additionally, healthy dietary patterns provide beneficial fats (monounsaturated and polyunsaturated fatty acids) that improve how the body responds to insulin. These healthy fats work by enhancing cellular energy production and reducing inflammatory stress in cells (41, 42).

A key finding of this study is the absence of a statistically significant association between adherence to the ‘processed food pattern' and GDM risk. This observation appears to contradict a substantial body of evidence, including meta-analyses (22) and large cohort studies (23, 37), that links ‘Western-style' dietary patterns with a markedly increased risk of GDM. We propose that our processed pattern did not reflect caloric excess typical of Western diets but rather indicated dietary lower with micronutrient intake. Typical southern Chinese diets emphasize natureal food such as rice, vegetables, and meat (25), and Western patterns are characterized by elevated consumption of processed meats, refined grains, and sugar-sweetened beverages with high energy density. The processed pattern in our Miao cohort represented an intermediate dietary stage: processed foods partially displaced nutrient-dense traditional items. However, the absolute intake of these processed foods was substantially lower than in Western diets (33, 42). This combination led to micronutrient deficiencies, potentially representing an early nutrition transition stage in pregnant women. This finding suggests that insufficient energy accompanied by micronutrient deficiency may be an independent GDM risk factor. Furthermore, pregnant women in this pattern may exhibit more severe pregnancy-related nausea or generally poor appetite. This could lead to reduced intake of regular meals (which are heavily salted) and substitution with small amounts of processed snacks and beverages. This interpretation is consistent with the overall pattern of dietary insufficiency we observed in this group, including lower total energy, protein, and micronutrient intake. Notably, this pattern retained protective traditional components, particularly sour soup, whose beneficial effects may have partially masked adverse associations from less-healthy elements.

We identified a novel inverse association between sour soup consumption and GDM risk, with restricted cubic spline analysis demonstrating an approximately linear dose-response protective relationship during preconception and early pregnancy. To our knowledge, this is the first epidemiological evidence linking this traditional Miao fermented condiment to GDM prevention. Sour soup is a traditional Miao fermented condiment made from tomatoes, chili peppers, and glutinous rice. The fermentation process creates beneficial bacteria, including lactic acid bacteria and yeast species (33). We propose that sour soup's protective effects against GDM work through improving the balance of bacteria in the mother's digestive system. Similar to how yogurt benefits health, the beneficial microorganisms in sour soup may help restore healthy gut bacteria during pregnancy (43). This improved bacterial balance may reduce pregnancy-related inflammation and help the body process blood sugar more effectively by supporting the insulin-producing cells in the pancreas (44–46).

Our study found a trend suggesting that pickled vegetable consumption might reduce GDM risk, though this association was not statistically significant. This finding deserves further investigation, as previous research from Guizhou, China, has reported similar protective trends for diabetes risk (47). Fermentation creates beneficial bacteria and compounds that may improve how the body responds to insulin by enhancing gut health (47, 48). Additionally, compounds such as luteolin and isoquercitrin-3-O-glucoside found in fermented vegetables have demonstrated inhibitory effects, suggesting a potential role in diabetes management (49). However, pickled vegetables have potential health concerns. Traditional fermentation methods may produce nitrites, which can form harmful compounds that increase cancer risk (47). Therefore, future research should focus on optimizing fermentation techniques and assessing the health benefits and safety of fermented pickled vegetables, particularly in the context of diabetes prevention and management.

Our study has several strengths. First, the prospective cohort design effectively mitigates the risk of reverse causality, thereby enhancing the reliability of our findings. Second, by assessing dietary intake both preconception and during early pregnancy, we were able to distinguish the independent associations between diet and GDM risk at these two distinct time points, providing valuable insights into the relative contributions of each period to GDM risk. However, there are several limitations to consider. Dietary intake data largely relied on retrospective self-reporting, which may introduce recall bias. Nevertheless, the FFQ used in this study has demonstrated relatively good reproducibility and acceptable validity in the Miao pregnant women, and we excluded participants with implausible energy intakes. Importantly, our findings indicate that dietary interventions for pregnant women should emphasize improving overall dietary quality and micronutrient density rather than caloric restriction alone. The protective association with traditional fermented foods warrants investigation of similar culture-specific fermented products (such as kimchi, tempeh, or injera) in other ethnic populations. However, direct generalizability is limited by our specific population characteristics, unique preparation methods of Miao traditional foods, and the rural low-income context. Future research should include multi-ethnic comparative studies, mechanistic investigations of traditional fermented foods across cultures, and randomized controlled trials evaluating culturally tailored dietary interventions to determine which protective principles are universal vs. population-specific.

## Data Availability

The raw data supporting the conclusions of this article will be made available by the authors, without undue reservation.
