# Correction: Pre-pregnancy and early pregnancy dietary patterns and gestational diabetes risk among Miao women in China

**DOI:** 10.3389/fnut.2026.1782266

**Published:** 2026-01-28

**Authors:** Song Zhang, Xiaorong Ni, Tian Qiao, Danqing Zhao, Liming Shen, Yi Liang

**Affiliations:** 1 Department of Endocrinology, Affiliated Hospital of Guizhou Medical University, Guiyang, China; 2 Department of Clinical Nutrition, Shenzhen Hengsheng Hospital, Shenzhen, China; 3 Department of Clinical Nutrition, Affiliated Hospital of Guizhou Medical University, Guiyang, China; 4 Obstetrics, Affiliated Hospital of Guizhou Medical University, Guiyang, China; 5 College of Life Science and Oceanography, Shenzhen University, Shenzhen, China

**Keywords:** dietary patterns, gestational diabetes mellitus (GDM), prospective cohort, pregnant women, Chinese Miao ethnicity

Author Song Zhang was erroneously assigned as corresponding author. The correct corresponding author is Yi Liang. The equal contribution and co-first authors statement was missing in the article as published. The authors, Song Zhang and Xiaorong Ni, should be designated as co-first authors who contributed equally to this work.

The original version of this article has been updated.

