# Supplementary material for: Pre-pregnancy and early pregnancy dietary patterns and gestational diabetes risk among Miao women in China
Source: Front Nutr. 2026 Jan 6;12:1663054. doi: 10.3389/fnut.2025.1663054 (PMC12815701; doi:10.3389/fnut.2025.1663054)
Supplement: Supplementary file 1 [file Table_1.docx]

Supplementary Material

# Supplementary Tables

**Table S1. Factor loading matrix of dietary pattern one year before pregnancy: Results of principal component analysis based on varimax rotation^1^**

| Food groups (g/day) | One year preconception | |  | Early pregnancy | |
| --- | --- | --- | --- | --- | --- |
|  | Prudent | Processed |  | Prudent | Processed |
| Refined grains | — | — |  | — | — |
| Beans | 0.48 | — |  | 0.43 | — |
| Whole Grains | 0.43 |  |  | — |  |
| Solanaceous Veg^2^ | 0.46 | — |  | 0.42 | — |
| Root Veg | 0.58 | — |  | 0.51 | — |
| Leafy Veg | 0.53 | — |  | 0.40 | — |
| Others Veg | 0.45 | — |  | 0.40 | — |
| Pickled Veg | — | — |  | — | — |
| Fungi/algae | 0.58 | — |  | 0.53 | — |
| Fruits | 0.46 | — |  | — | — |
| Dairy | — | — |  | 0.34 | — |
| Dairy Dessert | — | 0.40 |  | — | 0.63 |
| Livestock | 0.40 | — |  | 0.41 | — |
| Poultry | — | — |  | 0.31 | — |
| Organ | — | — |  | 0.54 | — |
| Processed Meat | — | 0.43 |  | — | 0.37 |
| Fish/Shrimp/Shellfish | 0.36 | — |  | 0.51 | — |
| Egg | 0.40 | — |  | 0.44 | — |
| Nuts | — | — |  | 0.34 | — |
| Snacks | — | 0.50 |  | — | 0.60 |
| Convenience Foods | — | 0.58 |  | — | — |
| Sour soup | 0.33 | — |  | — | 0.49 |
| Beverages | — | 0.61 |  | — | 0.42 |
| Wine | — | 0.36 |  | — | — |
| Explained variance（%） | 11.78 | 7.58 |  | 11.25 | 8.13 |

^1^The values represent factor loadings, which indicate the correlation between individual food item and dietary patterns identified through principal component analysis. Food groups are arranged in descending order of loading magnitude, with only coefficients having absolute values of 0.3 or greater displayed in the table. ^2^ Vegetable abbreviated as Veg.

**Table S2. Food group consumption among Miao pregnant women: comparison across dietary patterns during one year preconception and first trimester^1^**

| Food groups  (g/day) | One year preconception | | *P* value | Early pregnancy | | *P* value |  |
| --- | --- | --- | --- | --- | --- | --- | --- |
|  | Prudent | Processed |  | Prudent | Processed |  |  |
| n | | 329 | 354 |  | 345 | 338 |  |
| Refined Grains | | 304.14 ± 185.71 | 292.14 ± 163.81 | 0.223 | 271.43 ± 152.14 | 265.51 ± 133.61 | 0.182 |
| Beans | | 83.94 ± 115.24 | 67.98 ± 95.19 | 0.001 | 107.14 ± 140.85 | 86.71 ± 110.69 | 0.004 |
| Whole Grains | | 42.49 ± 59.18 | 37.51 ± 59.17 | 0.072 | 54.11 ± 77.73 | 31.76 ± 47.38 | <0.001 |
| Solanaceous Veg^2^ | | 65.00 ± 102.86 | 47.82 ± 70.46 | <0.001 | 60.71 ± 87.86 | 49.29 ± 76.43 | 0.005 |
| Root Veg | | 24.64 ± 37.33 | 11.00 ± 18.31 | <0.001 | 16.67 ± 36.11 | 12.28 ± 24.87 | <0.001 |
| Leafy Veg | | 266.14 ± 350.00 | 138.57 ± 181.07 | <0.001 | 227.14 ± 280.00 | 150.00 ± 228.14 | <0.001 |
| Others Veg | | 38.67 ± 68.24 | 23.90 ± 39.32 | <0.001 | 37.57 ± 63.00 | 26.19 ± 49.75 | 0.001 |
| Pickled Veg | | 1.00 ± 4.20 | 2.06 ± 8.35 | <0.001 | 0.67 ± 3.33 | 1.37 ± 4.29 | 0.022 |
| Fungi/algae | | 10.33 ± 17.93 | 7.73 ± 12.60 | <0.001 | 10.31 ± 18.12 | 5.71 ± 12.20 | <0.001 |
| Fruits | | 279.95 ± 280.17 | 188.01 ± 197.12 | <0.001 | 266.00 ± 269.50 | 236.33 ± 280.26 | 0.079 |
| Dairy | | 91.43 ± 214.28 | 71.43 ± 136.54 | 0.105 | 250.00 ± 152.14 | 98.45 ± 225.98 | <0.001 |
| Dairy Dessert | | 0.00 ± 6.00 | 3.00 ± 10.00 | <0.001 | 0.00 ± 0.00 | 0.00 ± 6.00 | <0.001 |
| Livestock | | 110.57 ± 144.20 | 101.76 ± 83.85 | <0.001 | 86.95 ± 94.57 | 51.39 ± 83.65 | <0.001 |
| Poultry | | 8.33 ± 18.10 | 6.67 ± 10.96 | 0.006 | 8.33 ± 13.34 | 3.33 ± 7.14 | <0.001 |
| Organ | | 0.50 ± 3.33 | 0.00 ± 2.50 | 0.213 | 0.75 ± 4.67 | 0.00 ± 1.17 | <0.001 |
| Processed Meat | | 1.32 ± 3.86 | 3.86 ± 9.70 | <0.001 | 0.40 ± 3.33 | 1.07 ± 5.11 | 0.002 |
| Fish/Shrimp/  Shellfish | | 15.96 ± 26.66 | 12.73 ± 22.87 | 0.176 | 16.95 ± 29.32 | 7.53 ± 14.83 | <0.001 |
| Egg | | 26.21 ± 51.10 | 12.92 ± 24.37 | <0.001 | 60.00 ± 30.00 | 17.14 ± 27.29 | <0.001 |
| Nuts | | 0.58 ± 3.75 | 0.75 ± 2.91 | 0.747 | 4.67 ± 16.30 | 1.29 ± 7.71 | <0.001 |
| Snacks | | 3.00 ± 10.97 | 9.62 ± 19.23 | <0.001 | 1.60 ± 6.53 | 5.39 ± 18.92 | <0.001 |
| Convenience Foods | | 9.05 ± 16.00 | 22.82 ± 43.50 | <0.001 | 12.50 ± 24.40 | 13.50 ± 26.00 | 0.61 |
| Sour soup | | 41.90 ± 100.96 | 40.00 ± 86.67 | 0.191 | 33.33 ± 78.57 | 34.29 ± 106.71 | 0.373 |
| Beverages | | 35.00 ± 98.28 | 114.51 ± 194.92 | <0.001 | 15.75 ± 50.00 | 40.54 ± 119.14 | <0.001 |
| Wine | | 1.10 ± 30.00 | 15.54 ± 87.80 | <0.001 | 0.00 ± 0.00 | 0.00 ± 0.00 | 0.033 |

^1^ Unless otherwise specified, data are presented as median ± interquartile range (IQR). *P*-values were calculated using the Wilcoxon rank sum test. ^2^ Vegetable abbreviated as Veg.

**Table S3. Food group consumption among Miao pregnant women: comparison across GDM diagnosis during one year preconception and first trimester**

| Food groups  (g/day) | | One year preconception | | *P* value | Early pregnancy | | *P* value |
| --- | --- | --- | --- | --- | --- | --- | --- |
|  |  | Non-GDM | GDM |  | Non-GDM | GDM |  |
| Refined Grains | 300.08 ± 167.93 | | 285.71 ± 210.91 | 0.372 | 273.76 ± 139.33 | 243.27 ± 145.77 | 0.005 |
| Beans | 76.43 ± 99.00 | | 77.72 ± 124.45 | 0.533 | 94.90 ± 122.32 | 83.91 ± 169.26 | 0.559 |
| Whole Grains | 41.05 ± 59.01 | | 37.71 ± 62.22 | 0.961 | 42.97 ± 60.85 | 32.31 ± 69.07 | 0.284 |
| Solanaceous Veg^1^ | 59.29 ± 86.29 | | 47.00 ± 83.12 | 0.033 | 56.00 ± 80.00 | 40.00 ± 69.02 | 0.003 |
| Root Veg | 16.00 ± 28.62 | | 17.88 ± 26.96 | 0.576 | 15.33 ± 28.81 | 14.29 ± 29.25 | 0.56 |
| Leafy Veg | 213.33 ± 253.43 | | 180.00 ± 205.36 | 0.53 | 178.57 ± 241.58 | 175.46 ± 220.25 | 0.984 |
| Others Veg | 28.83 ± 47.17 | | 35.98 ± 48.65 | 0.513 | 31.50 ± 55.33 | 37.14 ± 56.37 | 0.65 |
| Pickled Veg | 1.67 ± 6.11 | | 1.92 ± 4.01 | 0.873 | 0.98 ± 4.17 | 0.83 ± 3.33 | 0.427 |
| Fungi/algae | 8.73 ± 15.11 | | 9.85 ± 14.06 | 0.853 | 7.91 ± 16.07 | 5.64 ± 14.88 | 0.035 |
| Fruits | 234.00 ± 240.48 | | 198.48 ± 195.52 | 0.024 | 259.71 ± 266.95 | 213.73 ± 304.48 | 0.097 |
| Dairy | 85.71 ± 196.66 | | 45.42 ± 140.72 | 0.017 | 194.29 ± 195.24 | 176.19 ± 231.75 | 0.053 |
| Dairy Dessert | 0.86 ± 8.57 | | 0.00 ± 6.50 | 0.228 | 0.00 ± 2.33 | 0.00 ± 3.00 | 0.914 |
| Livestock | 104.00 ± 145.83 | | 102.80 ± 81.90 | 0.431 | 61.25 ± 84.08 | 60.21 ± 90.78 | 0.994 |
| Poultry | 7.14 ± 12.74 | | 7.14 ± 10.96 | 0.843 | 5.33 ± 11.19 | 7.14 ± 12.54 | 0.152 |
| Organ | 0.21 ± 2.50 | | 0.33 ± 2.46 | 0.801 | 0.00 ± 2.50 | 0.00 ± 1.92 | 0.271 |
| Processed Meat | 2.50 ± 6.66 | | 1.67 ± 6.16 | 0.107 | 0.80 ± 4.13 | 0.27 ± 3.33 | 0.171 |
| Fish/Shrimp/  Shellfish | 14.67 ± 26.14 | | 13.83 ± 22.04 | 0.732 | 11.33 ± 23.33 | 9.77 ± 18.56 | 0.591 |
| Egg | 19.14 ± 34.65 | | 19.21 ± 30.23 | 0.91 | 34.29 ± 47.14 | 34.29 ± 42.86 | 0.466 |
| Nuts | 0.67 ± 3.33 | | 0.59 ± 2.84 | 0.688 | 2.50 ± 12.00 | 2.60 ± 9.20 | 0.883 |
| Snacks | 6.17 ± 16.22 | | 3.63 ± 14.04 | 0.056 | 3.20 ± 12.04 | 1.97 ± 9.08 | 0.129 |
| Convenience Foods | 15.38 ± 28.67 | | 10.83 ± 22.81 | 0.036 | 14.29 ± 25.71 | 8.77 ± 20.34 | 0.009 |
| Sour soup | 41.90 ± 100.96 | | 32.95 ± 87.38 | 0.229 | 33.33 ± 95.53 | 30.95 ± 79.04 | 0.655 |
| Beverages | 76.91 ± 153.33 | | 40.25 ± 117.02 | 0.005 | 25.00 ± 85.00 | 13.81 ± 52.50 | 0.013 |
| Wine | 6.67 ± 57.14 | | 3.83 ± 51.90 | 0.754 | 0.00 ± 0.00 | 0.00 ± 0.00 | 0.913 |

^1^ Unless otherwise specified, data are presented as median ± interquartile range (IQR). *P*-values were calculated using the Wilcoxon rank sum test. ^2^ Vegetable and gestational diabetes mellitus are abbreviated to Veg and GDM, respectively.

**Table S4. Nutrients intake among Miao pregnant women: comparison across GDM diagnosis during one year preconception and first trimester**

| Nutrients | One year preconception | | *P* value | Early pregnancy | | | *P* value | |
| --- | --- | --- | --- | --- | --- | --- | --- | --- |
|  | Non-GDM | GDM |  | Non-GDM | GDM | |  |  |
| Energy, kcal d ^-1^ | 2045.56 ± 984.57 | 1807.48 ± 776.72 | <0.001 | 2045.56 ± 984.57 | | 1807.48 ± 776.72 | | <0.001 |
| Protein, g d ^-1^ | 64.48 ± 38.68 | 61.08 ± 34.37 | 0.067 | 64.48 ± 38.68 | | 61.08 ± 34.37 | | 0.067 |
| Fat, g d ^-1^ | 86.04 ± 47.29 | 76.44 ± 41.79 | 0.002 | 86.04 ± 47.29 | | 76.44 ± 41.79 | | 0.002 |
| SFA, g d ^-1^ | 24.42 ± 15.47 | 23.06 ± 13.48 | 0.043 | 24.42 ± 15.47 | | 23.06 ± 13.48 | | 0.043 |
| MUFA, g d ^-1^ | 27.90 ± 17.87 | 25.86 ± 14.59 | 0.06 | 27.90 ± 17.87 | | 25.86 ± 14.59 | | 0.06 |
| PUFA, g d ^-1^ | 12.03 ± 5.19 | 11.11 ± 5.24 | 0.012 | 12.03 ± 5.19 | | 11.11 ± 5.24 | | 0.012 |
| Carbohydrate, g d ^-1^ | 254.10 ± 134.06 | 222.70 ± 90.14 | <0.001 | 254.10 ± 134.06 | | 222.70 ± 90.14 | | <0.001 |
| Cholesterol，g d ^-1^ | 338.09 ± 288.06 | 309.27 ± 273.81 | 0.343 | 338.09 ± 288.06 | | 309.27 ± 273.81 | | 0.343 |
| Fiber，g d ^-1^ | 12.86 ± 9.03 | 11.64 ± 7.41 | 0.181 | 12.86 ± 9.03 | | 11.64 ± 7.41 | | 0.181 |
| Vitamin A, ug d ^-1^ | 1242.30 ± 1129.95 | 1163.82 ± 1166.96 | 0.369 | 1242.30 ± 1129.95 | | 1163.82 ± 1166.96 | | 0.369 |
| Thiamine, mg d^-1^ | 0.82 ± 0.54 | 0.76 ± 0.49 | 0.142 | 0.82 ± 0.54 | | 0.76 ± 0.49 | | 0.142 |
| Riboflavin, mg d^-1^ | 1.01 ± 0.59 | 0.94 ± 0.51 | 0.026 | 1.01 ± 0.59 | | 0.94 ± 0.51 | | 0.026 |
| Niacin, mg d^-1^ | 17.91 ± 11.65 | 16.64 ± 10.39 | 0.067 | 17.91 ± 11.65 | | 16.64 ± 10.39 | | 0.067 |
| Vitamin B6, mg d^-1^ | 0.15 ± 0.14 | 0.14 ± 0.13 | 0.132 | 0.15 ± 0.14 | | 0.14 ± 0.13 | | 0.132 |
| Vitamin D, mg d^-1^ | 1.57 ± 2.21 | 1.36 ± 2.12 | 0.28 | 1.57 ± 2.21 | | 1.36 ± 2.12 | | 0.28 |
| Vitamin C, mg d^-1^ | 597.79 ± 952.98 | 467.99 ± 824.57 | 0.048 | 597.79 ± 952.98 | | 467.99 ± 824.57 | | 0.048 |
| Vitamin E, mg d^-1^ | 22.61 ± 10.56 | 20.37 ± 10.57 | 0.009 | 22.61 ± 10.56 | | 20.37 ± 10.57 | | 0.009 |
| Folic acid, ug d^-1^ | 44.37 ± 46.51 | 42.04 ± 35.99 | 0.47 | 44.37 ± 46.51 | | 42.04 ± 35.99 | | 0.47 |
| Carotene, mg d^-1^ | 5637.44 ± 5198.65 | 5131.81 ± 5172.94 | 0.379 | 5637.44 ± 5198.65 | | 5131.81 ± 5172.94 | | 0.379 |
| Calcium, mg d^-1^ | 635.52 ± 421.35 | 554.53 ± 379.17 | 0.055 | 635.52 ± 421.35 | | 554.53 ± 379.17 | | 0.055 |
| Potassium, mg d^-1^ | 2705.22 ± 1682.25 | 2366.47 ± 1390.13 | 0.007 | 2705.22 ± 1682.25 | | 2366.47 ± 1390.13 | | 0.007 |
| Magnesium, mg d^-1^ | 302.33 ± 187.89 | 285.74 ± 156.66 | 0.098 | 302.33 ± 187.89 | | 285.74 ± 156.66 | | 0.098 |
| Iron, mg d^-1^ | 18.76 ± 10.43 | 16.85 ± 10.09 | 0.023 | 18.76 ± 10.43 | | 16.85 ± 10.09 | | 0.023 |
| Sodium, mg d^-1^ | 10560.08 ± 5032.52 | 11560.21 ± 5005.25 | 0.388 | 10560.08 ± 5032.52 | | 11560.21 ± 5005.25 | | 0.388 |
| Iodine, mg d^-1^ | 1.60 ± 1.76 | 1.28 ± 1.89 | 0.117 | 1.60 ± 1.76 | | 1.28 ± 1.89 | | 0.117 |

^1^ Unless otherwise specified, data are presented as median ± interquartile range (IQR). *P*-values were calculated using the Wilcoxon rank sum test. ^2^ Gestational diabetes mellitus abbreviated as GDM.
